# Editorial: Artificial Intelligence in Chemistry

**DOI:** 10.3389/fchem.2020.00275

**Published:** 2020-04-09

**Authors:** John C. Cancilla, José S. Torrecilla, Charalampos Vasilios Proestos, José Omar Valderrama

**Affiliations:** ^1^Scintillon Institute, San Diego, CA, United States; ^2^Departamento de Ingeniería Química y de Materiales, Universidad Complutense de Madrid, Madrid, Spain; ^3^Laboratory of Food Chemistry, Department of Chemistry, National and Kapodistrian University of Athens, Athens, Greece; ^4^Centro de Información Tecnológica (CIT), La Serena, Chile

**Keywords:** artificial intelligence, machine learning, artificial neural networks, deep learning, modern chemistry, chemical applications

Within our Research Topic, six unique manuscripts which contain different trained machine and deep learning algorithms to model chemical processes have been published. The optimized intelligent tools cover applications within several scopes including (i) chemical and physicochemical molecule property predictions, (ii) compound ranking, identification, and classification, (iii) monitoring and aiding drug discovery, as well as (iv) quality evaluation and classification of chemicals and foods.

In the successive paragraphs, each of the accepted publications are presented in chronological order and briefly described:

1. **e-Sweet: A Machine-Learning Based Platform for the Prediction of Sweetener and Its Relative Sweetness** (Zheng et al.). The authors of this work have designed and made available a free machine learning software platform called “e-Sweet” that predicts the relative sweetness of different molecules. They used a database containing the structures of many different compounds, both sweeteners and non-sweeteners, to train an array of machine learning models (e.g., support vector machine, random forest, or deep neural networks) that label each molecule tested with a relative sweetness value. Their aspiration is to empower food scientists to discover and develop new molecules with enhanced sweetness by harnessing the power of their intelligent platform.
2. **Deep Neural Network Classifier for Virtual Screening Inhibitors of (S)-Adenosyl-L-Methionine (SAM)-Dependent Methyltransferase Family** (Li et al.). In this study, the research team developed a deep learning-based neural network model to classify active vs. inactive compounds in relation to their ability to inhibit SAM-dependent methyltransferases. These targets are enzymes that possess a relevant epigenetic role and are pharmacologically significant as they are involved in the pathogenesis of several genetic disorders as well as cancer. To train their model, 12 unique targets (methyltransferases) were analyzed, using up to 1,740 different ligands (potential inhibitors) as samples to be classified, reaching improved statistical performances when compared to previous studies.
3. **Neural Networks Are Promising Tools for the Prediction of the Viscosity of Unsaturated Polyester Resins** (Molina et al.). Here, a neural network model was designed and optimized to determine a physicochemical property such as viscosity of unsaturated polyester resins, which are employed to synthesize composite materials. Viscosities are directly related to the performance of these materials, which leads to the intrinsic value of the accurate intelligent mathematical algorithm developed for the industry.
4. **Prediction of the Antioxidant Response Elements' Response of Compound by Deep Learning** (Bai et al.). During this research, the authors trained several deep learning algorithms to identify compounds that can hypothetically activate antioxidant response elements, which may lead to elevated toxicities linked to the appearance of oxidative stress. The strong performance offered by the team's optimized deep neural network (their most accurate model) implies the usefulness of machine learning to assess the safety of novel drugs and their future development, as molecules that potentially activate antioxidant response elements could be screened out.
5. **Development of Predictive Models for Identifying Potential S100A9 Inhibitors Based on Machine Learning Methods** (Lee et al.). In this work, the researchers analyzed a large dataset containing over six million compounds with the goal set to identify potential S100A9 inhibitors via machine learning algorithms including random forest classifiers. Their intelligent tool is relevant as S100A9 has been identified as a therapeutic target for various diseases including cancer and Alzheimer's, reason why facilitating the discovery of inhibiting drugs while vastly reducing costs is highly valuable for the field.
6. **Deep Learning Techniques to Improve the Performance of Olive Oil Classification** (Vega-Márquez et al.). In this final article, the authors cover the use of deep learning neural networks to classify olive oil samples in terms of quality by using data gathered via gas chromatography coupled to ion mobility spectrometry of over 700 samples to train the algorithms. The basic goal of their work is to reach tools that can help ensure the safety of olive oils in terms of health (being suitable for human consumption) and to avoid fraud (selling lower grade products as high quality ones).

These six articles are great examples which showcase the application of artificial intelligence in the shape of mathematical algorithms and machine learning to solve different technological and/or scientific problems of the chemical field. These manuscripts help readers understand the usefulness of these intelligent models and empowers them to design such tools to extract the most out of their experimental results to tackle problems of their own specific lines of research or technological development.

Artificial intelligence is offering alternatives and solutions that catalyze the creation and implementation of applications that would have been unconceivable or even impossible only 10 years ago. For this reason, many novel chemical industries, research projects, and ideas can greatly benefit from the inclusion of artificial intelligence, setting new frontiers while reaching a modernized and intelligent field of chemistry.

## Author Contributions

JC and JT wrote the editorial while CP and JV reviewed it.

### Conflict of Interest

The authors declare that the research was conducted in the absence of any commercial or financial relationships that could be construed as a potential conflict of interest.

